# Increase in the Antioxidant and Anti-Inflammatory Activity of *Euterpe oleracea* Martius Oil Complexed in β-Cyclodextrin and Hydroxypropyl-β-Cyclodextrin

**DOI:** 10.3390/ijms222111524

**Published:** 2021-10-26

**Authors:** Thalita Sévia Soares de Almeida Magalhães, Pollyana Cristina de Oliveira Macedo, Érika Cibely Pinheiro da Costa, Emanuella de Aragão Tavares, Valéria Costa da Silva, Gerlane Coelho Bernardo Guerra, Joquebede Rodrigues Pereira, Telma Maria de Araújo Moura Lemos, Marília Medeiros Fernandes de Negreiros, Hugo Alexandre de Oliveira Rocha, Attilio Converti, Ádley Antonini Neves de Lima

**Affiliations:** 1 Laboratório Escola de Farmácia Industrial, Department of Pharmacy, Federal University of Rio Grande do Norte, Natal 59012-570, Brazil; thalitasevia22@gmail.com (T.S.S.d.A.M.); macedopollyanax@gmail.com (P.C.d.O.M.); e.cibelyp@gmail.com (É.C.P.d.C.); 2 Centro de Biociências, Department of Biophysics and Pharmacology, Federal University of Rio Grande do Norte, Natal 59078-570, Brazil; emanuella_ta@hotmail.com (E.d.A.T.); vcs.biomed@gmail.com (V.C.d.S.); gerlaneguerra@hotmail.com (G.C.B.G.); 3 Laboratório de Pesquisa em Bioquímica Clínica e Experimental, Department of Análises Clínicas e Toxicológicas, Federal University of Rio Grande do Norte, Natal 59012-570, Brazil; joque.rodrigues@gmial.com (J.R.P.); telmaml@yahoo.com.br (T.M.d.A.M.L.); 4 Department of Biochemistry, Federal University of Rio Grande do Norte, Natal 59078-570, Brazil; marilia_negreiros16@yahoo.com.br (M.M.F.d.N.); hugo-alexandre@uol.com.br (H.A.d.O.R.); 5 Department of Civil, Chemical and Environmental Engineering, Pole of Chemical Engineering, Genoa University, I-16145 Genoa, Italy; converti@unige.it

**Keywords:** *Euterpe oleraceae*, inclusion complexes, antioxidant, anti-inflammatory

## Abstract

Reactive oxygen species (ROS) are aerobic products generated during cellular respiration, but in the case of oxidative stress, they become key factors in the development of inflammatory processes and chronic diseases such as diabetes and rheumatoid arthritis. In this work, *Euterpe oleracea* oil (EOO), as well as the complexes produced by slurry (S) and kneading (K), were analyzed for antioxidant capacity in vitro, while only the β-cyclodextrin complex obtained by kneading (EOO-βCD-K), which showed better complexation, was selected for anti-inflammatory assays in vivo. In the scavenging activity of OH·, the hydroxypropyl-β-cyclodextrin complex obtained by kneading (EOO-HPβCD-K) exhibited an activity 437% higher than the pure oil. In the paw edema assay, EOO-βCD-K reduced edema by 200% and myeloperoxidase (MPO) activity by 112%. In an air pouch model, this treatment showed a reduction in leukocyte, MPO, and Interleukin-1β (IL-1β) levels; meanwhile those of glutathione and IL-10 were increased, demonstrating its ability to potentiate the anti-inflammatory effect of EOO.

## 1. Introduction

The response to inflammation is an innate or adaptive immune process that allows the body to defend itself from pathogens [1]. Tissue damage, either chemical or physical, is potentially capable of triggering an acute response characterized by the accumulation of nuclear polymorphs, such as macrophages, neutrophils and mast cells, dendritic cells, Natural Killer cells, as well as cytokines and chemokines responsible for the modulation of inflammation [2]. While this response is considered a benefit for the body, a chronic response is triggered if the process is prolonged or inefficient [3]. Reactive oxygen species (ROS), such as the superoxide anion (·O_2_^−^) and hydroxyl (OH·) radicals, as well as non-radical species, such as hydrogen peroxide (H_2_O_2_) and oxygen singlet (^1^O_2_), are aerobic products normally generated during cellular respiration [4]. However, in the case of oxidative stress, they become a key factor in the progression of various inflammatory processes, as well as chronic inflammatory diseases and comorbidities, including rheumatoid arthritis and diabetes mellitus [5,6]. These species, produced by nuclear polymorphs during the host’s organism defense, are also responsible for the oxidation of cell signaling proteins, promoting endothelial dysfunction in the inflammation site [7].

Since primordial times, products of natural origin with anti-inflammatory activity have been used as an alternative therapy [8]. *Euterpe oleracea* Martius, commonly known as “açaí” is a palm tree that grows naturally in the Amazon region [9,10]. Its fruit gained popularity in North America and Europe due to its recognized antioxidant potential, while the oil obtained from the fruit is popularly consumed for its anti-inflammatory properties. The phytochemical compositions of these products are well described in the literature and justify their applicability [11,12,13,14,15,16]. In general, vegetable oils are hardly used by the pharmaceutical industry due to their low solubility and hydrolytic rancidity. However, cyclodextrins have emerged as an alternative capable of solving such problems due to their ability to form inclusion complexes [17,18,19]. These complexes allow: (a) increasing biological solubility, activity, and oxidative stability, (b) reducing photosensitivity and thermosensitivity, (c) masking undesirable organoleptic characteristics, (d) reducing the volatility and toxicity of some drugs, and (e) protecting the host molecule from gastrointestinal degradation [20,21,22].

This work aims at testing the in vitro antioxidant capacity of inclusion complexes of *E. oleracea* Martius oil (EOO) with β-cyclodextrin (βCD) and hydroxypropyl-β-cyclodextrin (HP βCD), previously prepared by slurry (S) and kneading (K) methods, and physicochemically characterized. Samples were submitted to the superoxide ion and hydroxyl radical scavenging assays, iron and copper chelating capacity, total antioxidant capacity, and reducing potential. In vivo carrageenan-induced paw edema (anti-inflammatory) and air pouch assays were performed utilizing the β-cyclodextrin inclusion complex obtained by kneading. The biological materials obtained from the inflammation were submitted for dosages of myeloperoxidase (MPO), interleukin-10 (IL-10) and interleukin-1β (IL-1β), glutathione (GSH), and leukocyte count.

## 2. Results

### 2.1. Antioxidant Activity of EOO, βCD, HPβCD, EOO-βCD, and EOO-HPβCD

#### 2.1.1. Hydroxyl Radical (OH·) Scavenging Activity

**Figure 1:**
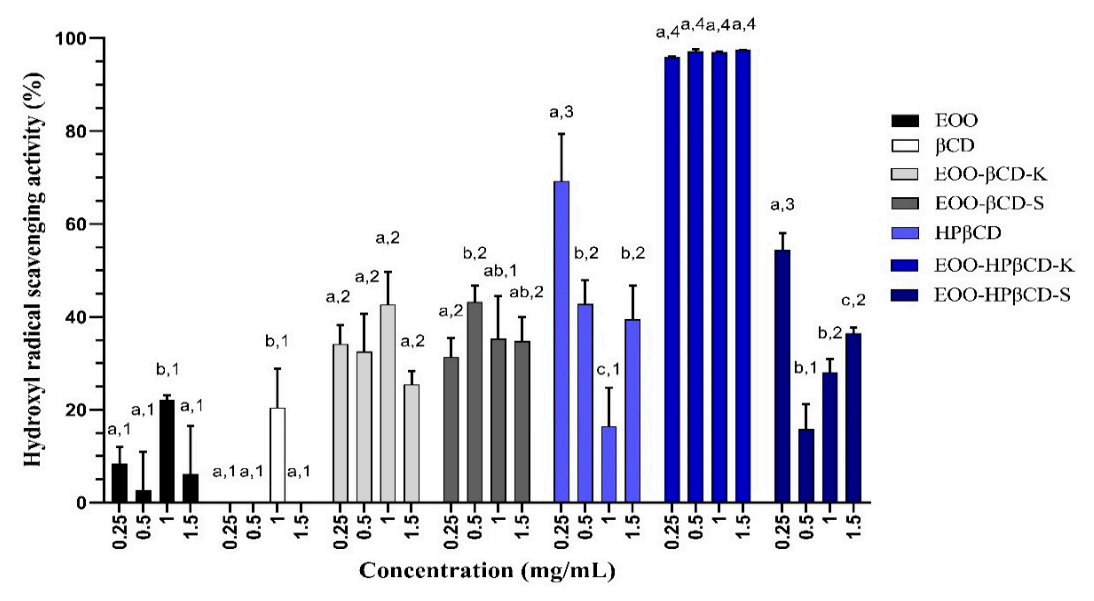
Hydroxyl radical scavenging capacity of EOO, βCD, HPβCD, and inclusion complexes. Methods used to prepare inclusion complexes: K = kneading; S = slurry. Different letters represent a significant difference between different concentrations of the same sample; different numbers represent a significant difference between different samples at the same concentration (*p* < 0.05).

EOO demonstrated 11.23%, 7.10%, 22.21%, and 8.5% of hydroxyl radical (OH·) scavenging activity at concentrations of 0.25, 0.5, 1.0, and 1.5 mg/mL respectively (Figure 1). This property does not appear to be dose-dependent since the 1.0 mg/mL sample achieved a significantly higher value than the others.

While the OH· scavenging activity of βCD (22.85%) at a dose of 1.0 mg/mL showed no significant difference compared to that of EOO at the same concentration, those of EOO-βCD obtained by kneading (EOO-βCD-K) (46.82%) at a dose of 1.0 mg/mL and by slurry (EOO-βCD-S) (46.62%) at a dose of 0.5 mg/mL were approximately 210% and 180% higher. HPβCD showed an even greater scavenging activity (74.94%) at a dose of 0.25 mg/mL, with no statistically significant difference compared to βCD complexes (Figure 1), which suggests this cyclodextrin has a good OH· scavenging potential. EOO-HPβCD complexes were the best in scavenging this radical, especially those prepared by kneading (97% activity with no dose influence), which allowed an activity increase of up to 437% compared to pure oil.

#### 2.1.2. Total Antioxidant Capacity (TAC)

**Figure 2:**
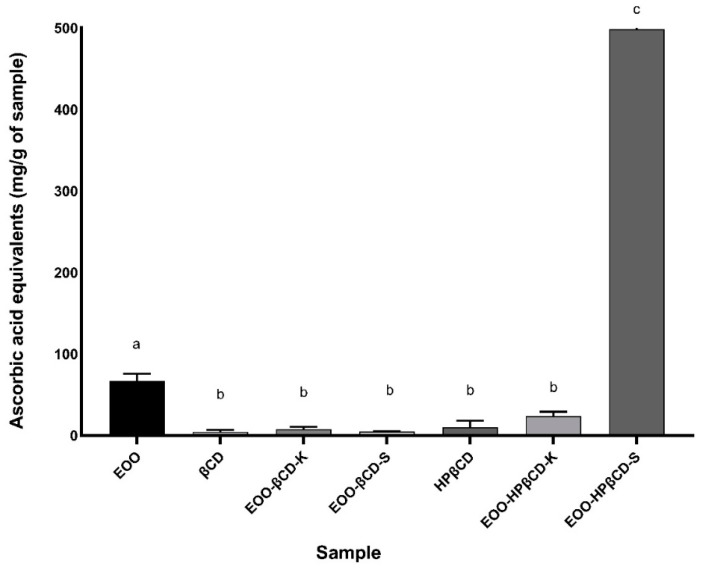
Total antioxidant capacity of EOO, βCD, HPβCD, and inclusion complexes. Different letters (a–c) represent statistically significant differences (*p* < 0.05).

All samples showed some degree of total antioxidant capacity, as shown in Figure 2. EOO exhibited a TAC of 66.84 mg of ascorbic acid equivalent per gram of sample. As for the TAC of the cyclodextrins, it is known that they tend to remain in a protonated state under an acidic environment, making it difficult to release H^+^ ions [23]. Thus, TAC values of 15.89 and 9.97 mg of ascorbic acid equivalent/g were considered acceptable for βCD and HPβCD, respectively. Although these mean values are different, their standard deviations prove that there is no statistically significant difference between them.

EOO-βCD complexes prepared by both methods, as well as EOO-HPβCD-K, showed no significant difference in the TAC response, with 7.45, 4.76, and 23.72 mg of ascorbic acid equivalent/g, respectively (Figure 2). These results suggest a reduction in EOO TAC, which can be explained, in the case of inclusion complexes, by the physicochemical changes that complexed molecules undergo and the consequent modification of the effects for which they are applied. Surprisingly, EOO-HPβCD-S exhibited a considerable donor activity, 498.28 mg of ascorbic acid equivalent/g, approximately 7.5 times higher than that of EOO. It is likely that the binding of CD with EOO allowed a complexation that induced a structural conformation capable of exposing internal OH groups responsible for the antioxidant activity.

#### 2.1.3. Reducing Power (RP)

**Figure 3:**
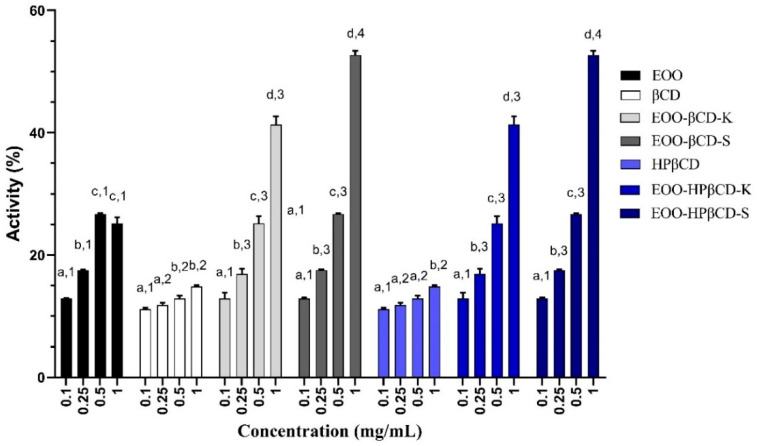
Reducing power of EOO, βCD, HPβCD, and inclusion complexes. Different letters represent a significant difference between different concentrations of the same sample; different numbers represent a significant difference between different samples at the same concentration (*p* < 0.05).

As shown in Figure 3, the reducing power of EOO was 12.90%, 17.54%, 26.73%, and 25.19% at concentrations of 0.1, 0.25, 0.5, and 1.0 mg/mL, respectively. Although the doses of 0.5 and 1.0 mg/mL did not differ significantly, EOO RP appeared to be dose dependent. For both cyclodextrins, RP values around 10–15% seem to show the same pattern as the pure oil. In some cases, RP showed a significant difference among the concentrations of the same sample but did not show a significant difference between the cyclodextrins.

The EOO-βCD-K at a dose of 0.1 mg/mL seems to have maintained the response of βCD with 12.90% activity, while at a dose of 0.5 mg/mL it practically maintained the EOO response with 25.19% activity at the same concentration. On the other hand, at a dose of 1.0 mg/mL it increased the RP by approximately 164% compared to EOO, exhibiting 41.34% activity. The EOO-βCD-S behaved similarly, although the dose of 1.0 mg/mL exhibited an even greater potentiating effect with an RP of 52.62%, which corresponds to an activity increase of approximately 208% compared to pure EOO. EOO-HPβCD complexes prepared by both methods showed similar responses to those produced with βCD, but in general they did not exhibit significant differences among samples and concentrations (Figure 3).

### 2.2. Anti-Inflammatory Activity of EOO and EOO-βCD Complex

#### 2.2.1. Effect of EOO and EOO-βCD in a Model of Paw Edema Induced by Carrageenan in Mice

**Figure 4:**
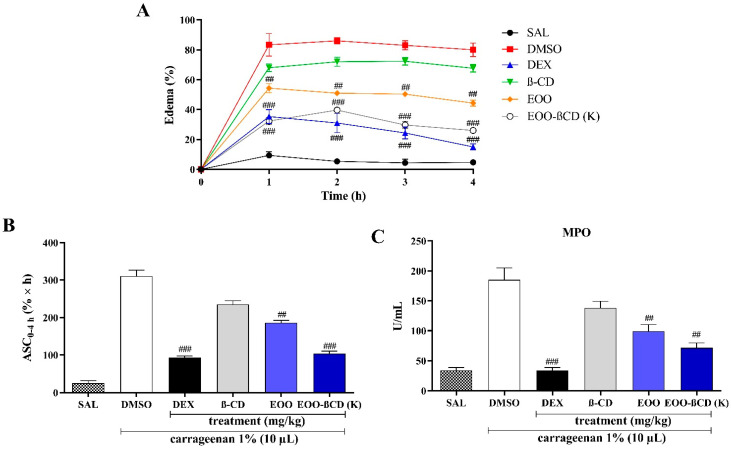
(**A**) Temporal evolution curve of edemas; (**B**) Area under the curve for the percentage of paw edema as a function of time (ASC0-4h); and (**C**) Myeloperoxidase activity. SAL: 0.9% saline solution; DEX: dexamethasone; DMSO: dimethyl sulfoxide. *p*-values for comparison with the positive control group (DMSO), after one-way ANOVA followed by Tukey’s test: ## *p* < 0.01 and ### *p* < 0.001.

The peaks of edema in mice paws occurred during the first hour after the carrageenan injection, persisting or progressively decreasing during consecutive paw measurements (Figure 4A). Subplantar injection of 1% carrageenan (10 µL/paw) induced a significant edematogenic effect in the first hours (*p* < 0.001) compared to the group treated with saline solution (negative control). As expected, the dexamethasone-treated group exhibited anti-inflammatory activity by reducing edema and tissue MPO activity.

The areas below the curves of edema evolution (ASC0-4h) show that the treatment performed with EOO (50 mg/kg, v.o.) led to a significant reduction in inflammation (*p* < 0.01) (Figure 4B), i.e., around 37% of the edema volume over the evaluated period compared to the dimethyl sulfoxide (DMSO) group. This response confirms an antiedematogenic activity previously reported by Favacho et al. [24]. Myeloperoxidase is an enzyme indirectly related to neutrophil migration and inflammation; therefore, if a given compound decreases its activity, it points to a potential anti-inflammatory effect [25]. Taking this into consideration, EOO also showed a significant anti-inflammatory effect, being able to reduce (*p* < 0.01) MPO activity in the DMSO group from 180 to 105 U/mL (Figure 4C).

In assessing the edematogenic response of EOO-βCD (300 mg/kg, v.o.), the sample improved the response of pure EOO (*p* < 0.001), reducing the edema volume by approximately 66% compared to the DMSO group (Figure 4A,B). The treatment with the inclusion complex led to a MPO activity of 68 U/mL, which demonstrates the inhibition (*p* < 0.01) of this enzyme (Figure 4C). Such an anti-inflammatory effect suggests a contribution to edema reduction, as shown in the ASC0-4h graphic. It is also noteworthy that EOO-βCD did not show a significant difference regarding the edematogenic response of the dexamethasone (DEX) group.

#### 2.2.2. Effect of EOO and EOO-βCD in a Model of Air Pouch Induced by Carrageenan in Mice

The inflammation in the mice air pouch model induced by carrageenan is an acute response characterized by intense cell migration, plasma exudation, and increase in total proteins towards the cavity of the animal’s back [26,27]. Therefore, in this study, we evaluated leukocyte migration, MPO activity, and the levels of glutathione (GSH), interleukin-1β (IL-1β), and interleukin-10 (IL-10).

**Figure 5:**
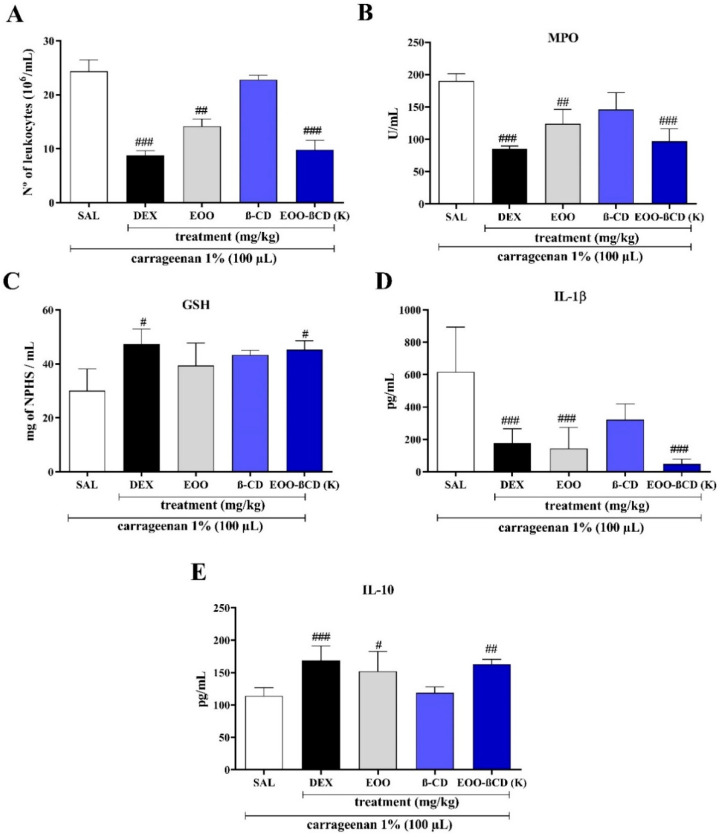
(**A**) Total leukocyte count; (**B**) Myeloperoxidase activity; (**C**) Glutathione level (GSH); (**D**) Interleukin 1-β activity; and (**E**) Interleukin-10 activity. *p*-values for comparison with the carrageenan group: # *p* < 0.05, ## *p* < 0.01, ### *p* < 0.001. Treatment with EOO and EOO-βCD-K in air pouch inflammation was induced by carrageenan. SAL: 0.9% saline solution; DEX: dexamethasone.

The use of both EOO (50 mg/kg, v.o.) and EOO-βCD-K (300 mg/kg, v.o.) significantly reduced the number of leukocytes compared to the saline solution (SAL) group, with the response of EOO-βCD (*p* < 0.001) being greater than that of EOO (*p* < 0.01) (Figure 5A). Consistently with this effect, MPO activity was reduced in the same treated groups (Figure 5B), suggesting the contribution on the reduction of inflammatory exudate. On the other hand, no statistically significant difference with the DEX group (Figure 5B) was observed in terms of both leukocytes number and MPO activity in paw edema.

GSH levels were measured, as this molecule is an important antioxidant that protects cells against damage induced by reactive oxygen species, removes hydrogen peroxide, and inhibits lipid peroxidation [28]. As shown in Figure 5C, the use of EOO did not show any difference in the GSH level when compared to the SAL group, while EOO-βCD-K significantly increased it (*p* < 0.05). This demonstrates an uptake of GSH from its oxidized form and considerable antioxidant protection during the inflammatory process, with an activity equivalent to the response shown by the DEX group.

Chen et al. [1] stated that by modulating the immune response to infection or inflammation, cytokines regulate it through a complex network of interactions. The use of EOO and EOO-βCD-K significantly reduced the IL-1β (*p* < 0.001) level compared to the SAL group (Figure 5C), while IL-10 levels increased in both the EOO (*p* < 0.05) and EOO-βCD-K (*p* < 0.01) groups, with greater significance of the latter response (Figure 5E).

## 3. Discussion

The unbalanced production of reactive oxygen species in living organisms is associated with various diseases, such as cancer, arteriosclerosis, inflammation, brain dysfunction, diabetes, neurodegenerative diseases, and premature aging [29,30]. Therefore, in vitro studies can be developed to discover compounds capable of acting as antioxidants [31]. According to Treml and Šmejkal [32], the sequestration of the hydroxyl radical (OH·) occurs through direct capture or extinction. These authors indicated three main mechanisms of reactions involving: (a) addition by inserting the radical into the molecule through double bonds, (b) electron transfer, and (c) hydrogen release, with consequent formation of water. Therefore, all reactions lead to the formation of new radicals and the propagation of chain reactions.

In OH· radical scavenging tests, EOO showed some activity, confirming the results of Pacheco-Palencia et al. [11]. These authors also highlighted the important antioxidant activity of phenolic acids and procyanidins retained in the EOO, considering it as a promising alternative to traditional oils for food, supplements, and cosmetic applications. A study carried out by Cao, Sofic, and Prior [33] on the antioxidant activity of a group of secondary metabolites attributed the OH· radical scavenging activity to the number of hydroxyl substituents and their position in the molecules, in particular, for anthocyanins, to C-3, C-3′, C-4′, C-5, and C-7 positions. In addition, Bendini et al. [34] indicated phenolic acids and the catechol moiety of many biomolecules as an important factor for this activity. Londhe et al. [35], in describing absorption spectra, attributed the OH· radical scavenging activity of different flavonoids to the presence of these same substituent groups [36], which are also present in EOO.

Our results indicate that the OH· radical scavenging capacity of the oil was improved after its complexation with cyclodextrins (CDs). It is believed that OH· can have interacted with oxygens of CDs, favoring free radicals’ stability. According to Jullian et al. [37], it is possible that free radicals become effectively stable in the CD cavity. A second hypothesis is related to the deprotonation capacity of cyclodextrins in an alkaline environment [23], allowing hydrogen atoms to neutralize the radicals. Furthermore, in agreement with the results of Li et al. [38], the OH· radical scavenging activity of HPβCD and its respective complexes was shown to be higher than that of βCD and its complexes, suggesting that the greater number of hydroxyl substituents on HPβCD allowed neutralization of these radicals more effectively.

As for the total antioxidant capacity (TAC) and reducing power (RP), only one article was found on EOO. Experiments carried out by Pacheco-Palencia et al. [36] proved the capacity of electrons or hydrogen atoms donation against the 2,2′-(3-ethylbenzothiazoline-6-sulfonic acid (ABTS+·) radical cation, the ferric reducing antioxidant power (FRAP), the 2,2-diphenyl-1-picrylhydrazyl (DPPH·) radical reduction, and oxygen radical absorption capacity (ORAC), inferring an important activity of the previously mentioned metabolites. Our most interesting results concerned the antioxidant responses in the different tests. Briefly, the use of EOO exhibited an important antioxidant activity in most tests carried out, especially those in which the electron-donating capacity (TAC and RP) was evaluated. Nonetheless, βCD and HPβCD proved to be interesting compounds in terms of RP and sequestration of OH· radicals, suggesting that in these assays not only scavenging of radicals occurred but also their elimination by hydrogen donation.

Complexes with CDs gave different responses. In general, although they showed low or no iron and copper chelating activity and superoxide ion scavenging (data not shown), OH· scavenging activity in the TAC and RP assays was definitely improved. EOO-HPβCD-K exhibited higher OH· scavenging activity compared to the oil alone. As for TAC, EOO-HPβCD-S stood out for exhibiting activity equivalent to half that of ascorbic acid, a result hardly found in other compounds. On the other hand, in the RP assay, complexes prepared with both cyclodextrins and methods showed a synergistic effect between EOO and CDs when the highest concentration was tested. The results also showed that not all ingredients or complexes exerted antioxidant activity in all tests. Such an activity sometimes depended on the type of cyclodextrin, the preparation process, and the tested dose. This can be important when specific mechanisms of action of the oxidative process must be blocked, for example, preventing lipid oxidation of biological membranes after the Fenton or Haber–Weiss reaction to generate OH·. EOO-HP-βCD-K stood out for its significant capacity to scavenge this radical.

Regarding the anti-inflammatory activity, in order to minimize the number of animals to be sacrificed, only the EOO-βCD-K system was tested, as it showed better energy of interaction in physicochemical assays and the kneading method better sample amorphization in a previous study [16]. A study by Favacho et al. [24] on the anti-inflammatory activity of EOO was found in the literature, in which the authors reported a significant reduction in carrageenan-induced paw edema. Although its positive control group was treated with indomethacin (10 mg/kg), instead of carrageenan as in the present study, the anti-inflammatory response of EOO in the tested dose (1226.8 mg/kg, v.o.) showed no significant difference compared to the positive control. On the other hand, no studies on the anti-inflammatory potential of EOO in an inclusion complex were found. It is known, however, that cyclodextrins, in addition to improving the physicochemical stability of drugs, can preserve or improve their activity [25,39,40,41]. This was also observed in the present study, when EOO-βCD-K increased the anti-hematogenic activity compared to pure EOO. A similar result was reported by Rodrigues et al. [41], who observed a significant inhibition of paw edema after administering *Ocimum basilicum* oil inclusion complexes with βCD.

The use of either EOO or EOO-βCD-K reduced the activity of MPO, providing results statistically coinciding with those obtained with dexamethasone. Since MPO is an enzyme present in abundance in neutrophil granules and, after activation, is released into the phagosome or extracellular space, it is considered a marker for the infiltration of these cells in the inflammation site [42,43,44]. According to Hasnat et al. [45], the decrease in MPO levels can be interpreted as an anti-inflammatory effect of a compound. Note that the MPO activities in both in vivo models used agree with the antiedematogenic activity (Figure 4A,B) and reduced leukocyte number (Figure 5A). It can, therefore, be concluded that EOO played a fundamental role in the anti-inflammatory action of the enzyme. In addition, the use of EOO-βCD-K in an air pouch model showed an even greater reduction in MPO activity compared to EOO alone. De Moura et al. [46] reported that the MPO activity was maintained using a hydroalcoholic extract (300 mg/kg/5 days) of the *Euterpe oleracea* fruit during the treatment of bronchoalveolar inflammation and continued exposure to cigarettes. These authors attributed the effect to the presence of previously identified proanthocyanidins. Pinheiro et al. [25] reported that the use of an inclusion complex of *Copaifera multijuga* oil with βCD also maintained the oil’s MPO activity.

The use of EOO in an air pouch model showed important activity in reducing leukocyte migration and ensuring favorable levels of GSH, IL-1β, and IL-10. The reduction in leukocyte migration confirms what was already observed in a similar study. In evaluating leukocyte migration in carrageenan-induced peritonitis, Favacho et al. [24] demonstrated that EOO (1226.8 mg/kg/d, v.o.) has an important effect in inhibiting these cells. However, the results obtained in the present study show that the use of EOO-βCD-K reduced the number of leukocytes more effectively than the pure oil. As for the GSH level, EOO showed no significant difference compared to the SAL group, while EOO-βCD-K showed similar values to the DEX group. When GSH levels are adequate, this antioxidant mechanism appears to be stable and to ensure a good recovery capacity of the injured tissue [47,48]. In this sense, it is suggested that the use of the complex contributes significantly to the antioxidant protection that occurs during an inflammatory process.

Note that the use of EOO and EOO-βCD-K contributed to favorable levels of cytokines involved in the inflammatory process. IL-1β induces the expression of adhesion molecules, such as selectins and integrins, which are essential for leukocyte chemotaxis to the inflammation site [49,50], while IL-10 acts in opposition to pro-inflammatory cytokines, induces the production of IL-1 Antagonist Receptor (IL-1RA), and releases Soluble Tumor Necrosis Factor Receptors (sTNFR), thereby limiting pro-inflammatory activities of IL-1 and Tumor Necrosis Factor (TNF) [51]. The IL-1β level decreased while that of IL-10 increased, confirming anti-inflammatory activity in an air pocket model.

## 4. Materials and Methods

### 4.1. Materials

The *Euterpe oleracea* Martius (açai) oil was obtained from Amazon Oil (Belém, Brazil), which reports that the açai fruits were collected in the Brazilian Amazon rainforest. The EOO, obtained from the fruit pulp, was extracted by a cold-pressing method without the addition of solvents or preservatives. β-cyclodextrin (βCD) and hydroxypropyl-β-cyclodextrin (HPβCD) were purchased from Sigma-Aldrich (St. Louis, MO, USA). Inclusion complexes were previously prepared by the kneading and slurry methods and characterized as previously described [16]. All the experiments were performed using purified water (<1.3 μS) obtained by reverse osmosis, and the reagents were of analytical grade.

### 4.2. In Vitro Antioxidant Activity of EOO, EOO-βCD and EOO-HPβCD

EOO, EOO-βCD, and EOO-HPβCD were diluted with a 10 mg/mL solution of dimethyl sulfoxide (DMSO) for analysis. The samples were evaluated for antioxidant activity by determining the superoxide radical scavenging activity, hydroxyl radical scavenging activity, iron (Fe^2+^) chelating activity, copper (Cu^2+^) chelating activity, total antioxidant capacity (TAC), and reducing power (RP). The concentrations used were 100, 250, 500, 1000, and 1.5 mg/mL. The methodologies were previously described by Presa et al. [52].

### 4.3. In Vivo Anti-Inflammatory Activity of EOO-βCD

Only EOO-βCD-K was chosen for the in vivo test because it exhibited better complexation parameters in physicochemical tests in previous work [16].

#### 4.3.1. Animals

Swiss mice (*Mus musculus*) of both sexes (females being nulliparous and non-pregnant), aged 6 to 8 weeks, and weighing from 25 to 35 g, were randomly divided into groups with five animals per group. They were kept under controlled lighting conditions (12 h light/dark cycle) and temperature (23 ± 2 °C), receiving water and food ad libitum. The experimental protocol number 031/2019 was approved by the Ethics Committee on the Use of Animals (CEUA) of the Federal University of Rio Grande do Norte (Natal, Brazil). All the animals submitted to tests were euthanized with an overdose of thiopental (150 mg·kg^−1^, intraperitoneal) and 2% lidocaine (10 mg·kg^−1^, intraperitoneal) at the end of the experiment.

#### 4.3.2. Carrageenan-Induced Paw Edema Test

Paw edema was induced by carrageenan as described by Winter et al. [53], with slight changes regarding the paw measurement period. To compare the responses of pure and complexed oil, a mass of oil equivalent to its concentration in the inclusion complex was selected. The oil, complexes, and carrageenan were then diluted in 500 μL (q.s.p.) of a 5% DMSO solution (*w/w*) as a vehicle. The right hind paw of all animals was measured with a digital caliper (Digimess, São Paulo, Brazil), next the mice were treated orally (v.o.) according to the following groups: EOO (50 mg·kg^−1^, v.o.), βCD (300 mg·kg^−1^, v.o.), EOO-βCD (300 mg·kg^−1^, v.o.), DEX (dexamethasone 2 mg·kg^−1^, v.o.), DMSO (5% DMSO, 500 μL, v.o.), and SAL (0,9% saline solution 500 μL, v.o.). The EOO dose was chosen based on previously developed dose-effect studies (results not shown). Paws received a 10 µL subplantar injection of 1% λ-carrageenan as an inflammatory agent, except for the negative control group. Paw size was measured 1, 2, 3, and 4 h after the administration of λ-carrageenan. Edema was expressed as the percentage of the difference between the paw size after (at respective time points) and before (basal values) carrageenan injection. The areas under the time-course curves (AUC0–4 h) were calculated using the trapezoidal rule [25]. The injured paw tissue was collected and processed according to Pinheiro et al. [25], and the pellets were used to extract and quantify MPO (see Section 4.3.4) as an indirect measure of neutrophils.

#### 4.3.3. Carrageenan Air Pouch

The air pouch model was developed according to Yoon et al. [54], with modification of the inflammatory agent, using λ-carrageenan. The randomized animals received, subcutaneously, 5 mL of sterile air on their back to form the pouch. After three days, the pouch was reinforced with 2.5 mL of sterile air. Six days after the first application, the animals were treated orally according to the specified group: EOO (50 mg·kg^−1^, v.o.), βCD (300 mg·kg^−1^, v.o.), EOO-βCD (300 mg·kg^−1^, v.o.), DEX (2 mg·kg^−1^, v.o.), and SAL (0.9% saline solution, v.o.). Furthermore, 100 µL of 1% λ-carrageenan was injected into the air bag. After six hours, the animals were euthanized, and the exudate in each air bag was collected after administration, followed by aspiration of 2 mL of sterile 0.9% saline solution. The biological material was centrifuged in an NT 805 mini centrifuge (Novatecnica, Piracicaba, São Paulo, Brazil) at 1500 rpm, 4 °C for 10 min, and the supernatant destined for determinations of cytokines (Section 4.3.5), interleukins 1-β (IL-1β) and 10 (IL-10), as well as glutathione (GSH) (Section 4.3.6) and MPO activity (Section 4.3.4). The cell pellet was used to count leukocytes in a Neubauer chamber after dilution in Turk’s solution.

#### 4.3.4. Myeloperoxidase Activity (MPO)

Myeloperoxidase activity was performed according to the method described by Krawisz, Sharon, and Stenson [55]. The samples obtained from the exudate of the air pouch were centrifuged to remove the cell pellet, then resuspended and homogenized with a micro-homogenizer (MA1102, Marconi, Piracicaba, Brazil) in a hexadecyltrimethylammonium bromide (HTAB) buffer in a 1:20 (*v*:*v*) ratio and subjected to ultrasonic irradiation (L200, Schuster, Santa Maria, Brazil) for 5 min. After that, the samples were centrifuged at 8285 rpm for 10 min at 4 °C. A total of 50 μL of the supernatant obtained from the homogenate was added to 150 μL of the staining reagent (*o*-dianisidine dihydrochloride, potassium phosphate buffer, and 1% hydrogen peroxide) and evaluated at 450 nm absorbance in a microplate reader (MR-96A, Mindray, Cidade Monções, Brazil). The results were interpolated with a standard curve of human neutrophil myeloperoxidase. The results were presented as U/mL of exudate, with one unit of MPO activity being the amount of enzyme capable of degrading 1 mmol of hydrogen peroxide/min at 25 °C.

#### 4.3.5. Cytokine Dosage—Interleukins 1-β (IL-1β) and 10 (IL-10)

The levels of IL-1β and IL-10 in the inflammatory exudate were measured using an enzyme-linked immunosorbent assay (ELISA) kit (R&D Systems Minneapolis, MN, USA). The exudate was centrifuged in the above mini centrifuge at 4285 rpm for 10 min at 4 °C, and the supernatant used to determine the absorbance in the above-mentioned microplate reader at 450 nm. The results were expressed in pg·mL^−1^.

#### 4.3.6. Glutathione (GSH)

The determination of GSH by the quantification of non-protein sulfhydryl radicals (NPSH) was carried out according to Sedlak and Lindsay [56]. A total of 320 μL of distilled water and 80 μL of 50% trichloroacetic acid were added to 400 μL of the inflammatory exudate, and the suspension was centrifuged at 3000 rpm (NT 805, Novatecnica) for 10 min at 4 °C. After processing, 100 μL of the supernatant was transferred to the reading plate along with 200 μL of Tris buffer—pH 8.9 (Tris + 0.2 M EDTA, q.s.p. H_2_O). To quantify NPSH in the sample, 5,5′-dithiobis (2-nitrobenzoic acid) (DTNB) was added, and the absorbance was read at 405 nm (MR-96A, Mindray). GSH levels were expressed in mg of NPHS/mL of the inflammatory exudate.

#### 4.3.7. Statistical Analysis

The results were presented as mean ± standard mean error. The antioxidant tests were carried out in triplicate and evaluated by one-way analysis of variance (ANOVA), followed by the Student Newman–Keuls test. In vivo experiments dealt with 5 animals per group, and the mean values were compared by one-way analysis of variance (ANOVA) followed by Tukey’s test or two-way ANOVA followed by Bonferroni’s test. For both, the GraphPad Prism version 6.00 (GraphPad Software, San Diego, CA, USA) was used. Values of *p* < 0.05 were considered significant.

## 5. Conclusions

In general, inclusion complexes with cyclodextrins (CDs) increased the effects of *Euterpe oleracea* oil (EOO). In the OH· radical scavenging test, βCD complexes prepared by kneading (K) at a dose of 1.0 mg/mL and by slurry (S) at a dose of 0.5 mg/mL showed 46.82% and 46.62% activities, respectively, which corresponded to 210% and 180% activity increases compared to EOO alone. Meanwhile, the activity of HP-βCD-K at a dose of 0.25 mg/mL was the highest (74.94%), with a 437% improvement compared to EOO alone. EOO-HP-βCD-S displayed a total antioxidant activity (TAC) of approximately 500 mg of ascorbic acid equivalent/g, with an effect 7.5 times greater than EOO, whereas other complexes were less effective than the oil alone. EOO-βCD-S increased the oil reducing power by 208%, while that prepared by kneading by 164%.

In the anti-inflammatory test of paw edema, EOO induced a reduction in local inflammation, showing a 37% decrease in edema volume and a reduction in myeloperoxidase (MPO) activity by approximately 42%. In this test, only the EOO-βCD-K inclusion complex was used since it showed a better complexation in previous characterization studies, in addition to exhibiting important hydroxyl radical scavenging capacity and reducing power. The results demonstrated that EOO-βCD-K potentiated the EOO effect, with a 66% reduction in the volume of paw edema and a 62% reduction in MPO activity, with no significant difference when compared to the dexamethasone-treated group.

Finally, in the air pouch assay, EOO-βCD-K significantly reduced the levels of leukocytes, MPO, and interleukin-1β, while interleukin-10 levels were increased. The MPO activity was 98 U/mL, which approximately corresponds to a 48% reduction compared to the result of the control group. This reduction corroborates with the response exhibited in the MPO test in paw edema. As for the GSH levels, whereas EOO showed no effect, EOO-βCD-K led to an increase in the levels of this marker.

## Data Availability

The data presented in this study are available on request from the corresponding author.
